# The right to information and their exceptions in medical practices in the Iranian legal system

**Published:** 2016-11-08

**Authors:** Maysam Sheykh-Talimi, Sadegh Shariati-Nasab, Mohammad Kazem Zare, Reza Omani-Samani

**Affiliations:** 1 Department of Epidemiology and Reproductive Health, Reproductive Epidemiology Research Center, Royan Institute for Reproductive Biomedicine, Academic Center for Education, Culture, and Research, Tehran, Iran;; 2 Research Supervisor, Department of Epidemiology and Reproductive Health, Reproductive Epidemiology Research Center, Royan Institute for Reproductive Biomedicine, Academic Center for Education, Culture, and Research, Tehran, Iran;; 3 PhD Candidate in Criminal Law and Criminology, University of Tehran, Tehran, Iran;; 4 PhD Candidate in Medical Ethics, Medical Ethics and History of Medicine Research Center, Tehran University of Medical Sciences, Tehran, Iran; Assistant Professor, Department of Epidemiology and Reproductive Health, Reproductive Epidemiology Research Center, Royan Institute for Reproductive Biomedicine, Academic Center for Education, Culture, and Research, Tehran, Iran.

**Keywords:** *Right to information*, *Access to information*, *Scope of access to information*, *Patients' information*, *Access to information exceptions*

## Abstract

The right to information Act was implemented in the Iranian legal system through accession of the Merida Convention ensuring the right to information as a fundamental right for the public. One significant aspects of this subject is the ratification of the "Disclosure and Access to Information Act" by which it is recognized as a right of all Persian individuals and citizens to access state-held information in Iran administration.

The Iranian legislature, with regard to the role of access to information and its significance, clarified the scope, permitted subjects of access, and exceptions of the right to state-held information. In this essay, we will discuss the legal aspects and scope of ensuring access to medical information in the Iranian statutes and their exceptions. It is argued that the Iranian legislation ensures the principle of maximum disclosure, while sensitive subjects’, specially classified and private information, are exempted. Therefore, the related rules in Iran’s statutes not only do not threaten patient’s information, but also protect them by criminalizing the breaching of the mentioned right.

## Introduction

The right to state-held information can be defined as the right of members of the public to know what state knows and accordingly state obligation to disclose information (1, 2). This Act was implemented in the Iranian legal system by accession to the United Nations Convention against Corruption (Merida).

Chapter II of the convention recognizes the right to access to information (3). Articles 10 and 13 of the mentioned chapter explicitly oblige state parties to ensure that the “public has effective access to information”. Consistent with provisions of the Merida Convention, state parties are obliged to report information to the public without any request from them and ensure their effective access to information.

By ratification of the “Disclosure and Access to Information Act” in August 2009, the legislature in Iran recognized the right of access to state-held information for members of the public and presented the subjects and scope of disclosure. The Iranian legislature then ratified the “Raising Health in Administration Body and Corruption combating Act” in November 2011. In this Act, preventive measures against corruption are the main purpose according to its provision, which predicted subjects of information dissemination and their exceptions.

In the mentioned Acts, disclosure before filing a request and requesting before disclosure of accessible information have been considered. On the one hand, the state is obliged to disclose information and the public has the right to file a request for retrieving information from public bodies. On the other hand, sensitive medical issues are under protection to respect the privacy of patients, except under special circumstances in which social benefit shall be achieved by disclosure of such information or personal information as discussed (4, 5). 

The main purpose of this essay was the description of the citizen’s right to access to public information and the clarification of patient rights and their exceptions in the area of right to information.

**Ensuring the right to information in the Iranian legal system**

The outset of ensuring the right to access to state-held information in Iran was the accession to the United Nations Convention against Corruption (Merida Convention).

Not too long after the accession, two significant Acts (6, 7) which ensure access to information with preventive approaches for fighting against corruption and increasing transparency were passed through the Iranian legislature body.

Thus, the obligation to ensure access to information established by the accession of the Merida Convention, and then, its recognition in the mentioned ratified Acts after the accession shall be discussed in this article.

**Obligation to ensure access to information**

Chapter II of the Merida Convention, which is the first and only international treaty for combating corruption, recognizes and declares the obligation of state parties to ensure access to information.

Article 5 of the convention mandates state parties to “develop anti-corruption policies that promote the participation of society” and “periodically evaluate legal instruments and administrative measures with a view to determining their adequacy to prevent and fight corruption”.

A variety of different measures are highlighted in chapter II, among which are recognition and ensuring of the right of access to state-held information. Provisions of article 10 and 13, respectively, consider the obligation of the state to disclose information actively and ensure effective access to information, both of which constitute the elements of the “right to state-held information”. In other words, there are two ways for accessing state-held information; individuals request to access information, and receiving it, or information is disseminated voluntarily by the state without any request from a user (8). Article 10 of the Merida Convention obliges state parties to “public reporting” which is the latter sort of access to information. Therefore, the state parties’ obligation is not only limited to accept file requests from users, but also includes reporting information proactively.

Article 13 of the convention, with the purpose of promoting active participation of individuals and groups outside the public sector, mandates state parties to promote transparency, participation of the public in the decision-making process, and effective access to information (9).

In fact, the article 13 is a repetition for ensuring access to information in order to recognize the right of access to information by individuals’ direct request without which the repetition of the right to information in article 13 could be unnecessary.

Ensuring the right to information has some fundamental demands without which access would be impossible; infrastructures and access without any discrimination for all members of the public. Thus, recognition of the right to information, and then, construction of primaries are state parties’ obligations. Therefore, article 10 and 13 together constitute the obligation to disclose information before and after any request, which are elements of the right of access to information.

**Ensuring the right to information in the Iranian statutes**

In response to the obligation to ensure the right to information in basis of articles 10 and 13 of the Merida Convention, which this convention is now a part of the Iranian legal system, as article 9 of the civil law adjudicate “ratified treaties (conventions) in parliament are a part of domestic law”, legislature recognized the mentioned right in Iran statute both in “Disclosure and access to Information Act” and “Raising Health in Administration Body and Combat Corruption Act” just after accession to the convention.

As mentioned above, these Acts, some parts of which are allocated to ensuring the right of access to information, have preventive approaches to combating corruption. As we can find at the chapeau of article 8 of the “Raising Health in Administration Body and Combat Corruption Act”, the legislature considers access to information for the public as an instrument for prevention of corruption.

For this purpose, the “Disclosure and Access to Information Act” observes that “each Persian individual has the right to access information”. Thus, article 5 in chapter 1 titled “Right to Information” states that “public bodies should disclose information which are subject to this Act as soon as possible and without any discrimination”. 

The right to state-held information as mentioned in the previous section of the text includes the right of individuals to file a request and obligation of the state to provide public reports. These elements can be found in article 2 and 5 of the “Access to Information Act”. Article 2 explicitly recognizes the right to information for all Persian people and article 5 holds that publishing the information is a duty for public bodies.

**Scope of access to state-held information in Iran**

To ensure access to information, according to the “Access to Information Act”, the state in a general notion is obliged to disclose all its information. Thus, the mentioned Act clarifies what information should be disclosed by public authorities in order to execute this right. It also considers exceptions of dissemination to clarify the duties of public bodies in order to perform disclosure rules precisely and for the purpose of protection of personal information.

*Subjects which must be disclosed*

In order to clarify public bodies’ duties, the “Raising Health in Administration Body and Combat Corruption Act” and “Disclosure and access to Information Act” explain what information can be disclosed. All information is accessible and inaccessibility is an exception; thus, the legislature in Iran named subjects which must be disclosed and their exceptions.

Article 5 of the “Disclosure and access to Information Act” holds public bodies responsible for the disclosure of information. However, article 10 states that “each public body should publish public information including at least their work results and functions annually in order to protect public benefit and citizenship rights using computer facilities and book guides…” and continues with naming some issues which must be disclosed by authorities.

It seems that topics which should be disclosed are not limited to the mentioned issues and “computer facilities” are not limited to filing a request before a public body, but publishing through the internet as a useful and costless way. 

In the “Raising Health in Administration Body and Combat Corruption Act”, several subjects of disclosure have been declared to clarify the duties of authorities to protect the right of accessing information. What should be disclosed in the latter Act can be considered as interpretation of the “Disclosure and Access to Information Act” which makes the subjects of access and exceptions more precise by explicitly declaring some subjects of access to information one by one such as proactively disseminating public contracts (article 3). But it does not make any limit through the accessible subjects, on the basis of “principle of maximum disclosure” which can be perceived by “Disclosure and Access to Information Act” (10) which means all information are accessible unless the law prohibits them to be disseminated. Thus, it seems that all medical information and statistics can be accessible unless an Act explicitly prohibits them. This at first glance seems contrary to the privacy and rights of patients to their information because these Acts, on the basis of maximum disclosure, make all information accessible unless an Act establishes an obstacle.

*Exceptions of access to state-held information*

Article 13 of the Merida Convention recognizes certain restrictions in the way of access to information. In Iran, as discussed above, the legislature have respected the principle of maximum disclosure, but, in order to respect privacy and public benefits, have banned access to some types of information. Section 4 of the "Disclosure and Access to Information Act" provides a number of exceptions including state secrets and classified documents, honoring privacy, health and commercial information protection, public security and tranquility, and etcetera. Therefore, the disclosure of sensitive information like name, address, personal habits, and body illness are explicitly excluded from disclosure and public access based on part (b) of article 1 of the "Disclosure and Access to Information Act". It is also imperative for authorities to refuse disclosure in case of the possibility of any harm or damage to life of individuals or their properties. Therefore, it seems that access to medical and sensitive information is restricted. One of the main exceptions to the right to information, as article 14 of the above Act states, is the request of access to information related to privacy of persons or reached by breaching their privacy. Preserving the privacy and confidentiality of patients is also a legal duty of medical healthcare providers (11, 12). Hence, as access to information ensures the fundamental rights of the public, exceptions to this right ensure trust between patients and healthcare providers, and protect the patient’s right to privacy and confidentiality. In other word, the other side of the same coin, is duty of healthcare providers, binding by article 648 of the Ta’azirat Act, which criminalized breaching confidentiality (13).

The above article not only provides an absolute prohibition of the disclosure of private information by all healthcare providers and other persons who have gained private information through their professions, but also makes it punishable by imprisonment or fine. Therefore, article 648 of the Ta’azirat Act is presumed as the criminal sanction of the “Disclosure and access to Information Act" restrictions. By this interpretation, the “Disclosure and Access to information Act” can be seen as a protective measure for patient’s medical information instead of a threat to it and article 648 of the Ta’zirat Act as a complementary to the mentioned restrictions.

As an exception to these rules, if the patient permits access through informed consent or if the patient has no legal capacity, the mentioned Act in article 15 prescribes the access of those who the patient permitted and patient’s guardians or attorney, respectively. It seems these exceptions to exceptions solve many legal issues in medical practices, which had remained unresolved and were an ethical dilemma for many years (14). 

However, according to the “Disclosure and Access to Information Act”, checking cases of exceptions is the duty of the “Access to Information Commission” in the Ministry of Culture of Iran, the purpose of which is ensuring the right of access to state-held information.

## Conclusion

The right to state-held information was implemented in the Iranian legal system by accession to the Merida Convention in the frame of ratification of the two significant Acts of the “Disclosure and Access to Information Act” and “Raising Health in Administration Body and Combat Corruption Act”. According to the first Act, access to information is recognized as a right for all Persian individuals and disclosure of information is mandatory for public bodies. The legislature by ensuring this right recognizes two ways of accessing information; voluntary dissemination of information by public bodies and disclosure by user request, which constitutes elements of the right of access to state-held information.

In the mentioned Acts, the legislature has declared what information shall be disclosed and has also determined their exceptions. In accordance with the Iranian legislature volition, disclosure is a general principle, while nondisclosure is presumed an exception. As a result, subjects of exceptions have been precisely determined in these Acts, and access to medical information is one of them. In the other words, in the era of freedom of information whereby the right to information ensures transparency in medical decision-making as a general rule, for the purpose of amelioration, its exception to patients information ensures privacy and confidentiality and trust between patients and healthcare providers. Medical information has been specifically exempted by the argued Acts from access, unless an Act gives permission to file a request for access; under some special and precise conditions such as informed consent or for the patients without legal capacity merely for their guardians or attorney.
